# Fractionation of Magnetic Microspheres in a Microfluidic Spiral: Interplay between Magnetic and Hydrodynamic Forces

**DOI:** 10.1371/journal.pone.0169919

**Published:** 2017-01-20

**Authors:** S. Dutz, M. E. Hayden, U. O. Häfeli

**Affiliations:** 1 Faculty of Pharmaceutical Sciences, University of British Columbia, Vancouver, Canada; 2 Institute of Biomedical Engineering and Informatics (BMTI), Technische Universität Ilmenau, Ilmenau, Germany; 3 Department of Physics, Simon Fraser University, Burnaby, Canada; Technion Israel Institute of Technology, ISRAEL

## Abstract

Magnetic forces and curvature-induced hydrodynamic drag have both been studied and employed in continuous microfluidic particle separation and enrichment schemes. Here we combine the two. We investigate consequences of applying an outwardly directed magnetic force to a dilute suspension of magnetic microspheres circulating in a spiral microfluidic channel. This force is realized with an array of permanent magnets arranged to produce a magnetic field with octupolar symmetry about the spiral axis. At low flow rates particles cluster around an apparent streamline of the flow near the outer wall of the turn. At high flow rates this equilibrium is disrupted by the induced secondary (Dean) flow and a new equilibrium is established near the inner wall of the turn. A model incorporating key forces involved in establishing these equilibria is described, and is used to extract quantitative information about the magnitude of local Dean drag forces from experimental data. Steady-state fractionation of suspensions by particle size under the combined influence of magnetic and hydrodynamic forces is demonstrated. Extensions of this work could lead to new continuous microscale particle sorting and enrichment processes with improved fidelity and specificity.

## Introduction

Magnetic microspheres (MMS) are employed in a variety of medical and pharmaceutical applications [1]. Typically they comprise superparamagnetic or ferrimagnetic iron oxide nanoparticles embedded in a spherical biodegradable polymer matrix. They often enclose a pharmaceutical agent [2, 3]. After injection into the blood stream of animals or humans, magnetostatic forces can be used to guide MMS to a target area where controlled release of the pharmaceutical agent occurs, acting on the target cells or disease [4, 5]. Most MMS used for drug delivery are coated with agents such as polyethylene glycols, which improve stability and blood circulation rates. The diameters of these particles are typically in the range 0.1 to 10 μm and exhibit a lognormal size distribution [6].

Efficient magnetic guiding of MMS is achieved using large particles, because the force acting on each particle is directly proportional to the volume of magnetic material it contains [7]. On the other hand, there is an absolute upper limit of about 5 μm on this diameter, which is imposed by the finite size of blood capillaries. Above this limit the risk of embolism leading to thrombosis and potential death of the patient is high [8]. Uniformity in the distribution of particle sizes is also desired; it is critical for reliable drug release from pharmaceutically-loaded MMS because kinetics of the release process depend on the surface-area to volume ratio [9]. Summarizing, MMS with uniform, controlled, and consistent sizes are needed to ensure optimal clinical magnetic targeting and defined drug release.

The preparation of biocompatible MMS fulfilling these stringent requirements is technically challenging; no suitable MMS are presently available from commercial sources. An alternate strategy is to prepare or acquire batches of MMS with broad size distributions, and subsequently apply size dependent fractionation techniques to obtain particles with the desired parameters.

Several methods for size-dependent fractionation of magnetic particles are described in the literature. They variously employ gravitational forces and centrifugation [10, 11], magnetostatic forces [12–15], hydrodynamic effects [16, 17], or electrostatic forces [18]. All suffer from the fact that they involve batch processing, and thus sample yield is limited.

This restriction can be overcome using microfluidic principles, which are compatible with continuous operation. Size-dependent fractionation has been accomplished in microfluidic systems based on filtration [19, 20], gravitational forces [21], sound pressure [22], diffusion [23], optical forces [24], and magnetostatic forces [25–29]. Summaries of developments in this area can be found in the reviews written by Pamme [30], Adams [31], Gossett [32], Sajeesh [33], and Shields [34].

Another established mechanism for achieving size-dependent fractionation of particles in microfluidic systems is the Dean effect [34–36]. This is an inertial effect that occurs when fluid flows through a curved channel. It amounts to the formation of a secondary flow (two or more counter rotating vortices) orthogonal to the primary downstream flow. When particles are present in the fluid stream, the combined action of hydrodynamic lift forces and the induced secondary flow causes particles that are larger than a critical (flow-dependent) size to migrate toward the inner wall of the channel. That is, toward the centre of curvature of the channel. The suitability of this effect for size-dependent fractionation of particles has been investigated through experiments in which particles of a certain size were focused or concentrated along a streamline [37], and in which fluorescent beads were extracted from human plasma [38] and filtered water [39]. The separation of neutrally buoyant particles into fractions of different mean size has also been demonstrated and shown to be consistent with numerical simulations [40–42].

In a previous report [43] we described and demonstrated the operation of a microfluidic spiral capable of size-dependent MMS fractionation based on the Dean effect and controlled by the fluid flow rate. Notably, we observed efficient fractionation of non-neutrally buoyant particles. We also observed efficient fractionation of MMS when a strong homogeneous magnetic field was applied. However, proper functioning of this device was severely degraded when magnetic field gradients were applied, causing particle agglomeration. This was particularly true near the inlet to the microfluidic spiral where flow rates (and hence hydrodynamic forces) were low, facilitating complete blockage of the channel.

The work described in our previous report sets the stage for experiments in which a carefully designed magnetic force, opposing the inwardly directed particle migration associated with the Dean effect, is applied to achieve enhanced control over the MMS fractionation process in a microfluidic spiral. In this paper we describe a magnetic structure that is suitable for this purpose, and investigate its influence on the fractionation process.

Our study reveals a new regime of stable particle focussing that is attributed to magnetostatic forces, and provides insight into the manner in which this equilibrium can be disrupted by curvature-induced hydrodynamic drag forces. It also reveals an experimental method for extracting quantitative in-situ information about hydrodynamic drag forces and local fluid velocities in microfluidic systems. From a microscale engineering perspective, our study lays the groundwork for the development of new devices for continuous sorting, manipulation, or concentration of MMS through simultaneous application of magnetic and curvature-induced hydrodynamic drag forces. In this context, it is worth noting that there are distinct advantages–in terms of flexibility, process fidelity, and specificity–to being able to leverage and control the interplay between two independent (competing or complementary) particle focusing or migration mechanisms, as opposed to relying on only one or the other.

## 1. Basic Principles

The continuous size dependent fractionation of MMS realized in our microfluidic chip results from the interplay between hydrodynamic and magnetostatic forces experienced by particles entrained in the laminar flow of a carrier medium through a microfluidic spiral. The key hydrodynamic effects include (a) drag forces induced by Dean vortices, (b) inertial lift forces associated with the action of a non-uniform flow profile on particles (shear effects), and (c) interaction of the flow field with the channel walls (particle wake effects). The magnetostatic effect is induced by a spatially non-uniform magnetic field that imposes an outwardly-directed (radial) force on particles at every point in the spiral. The combination of these effects leads to sorting of MMS by size across the radial dimension of the channel. Continuous separation of particles by size can then be achieved by splitting the flow into two or more discrete branches as it exits the spiral.

### 1.1 Hydrodynamic forces

**Fig 1:**
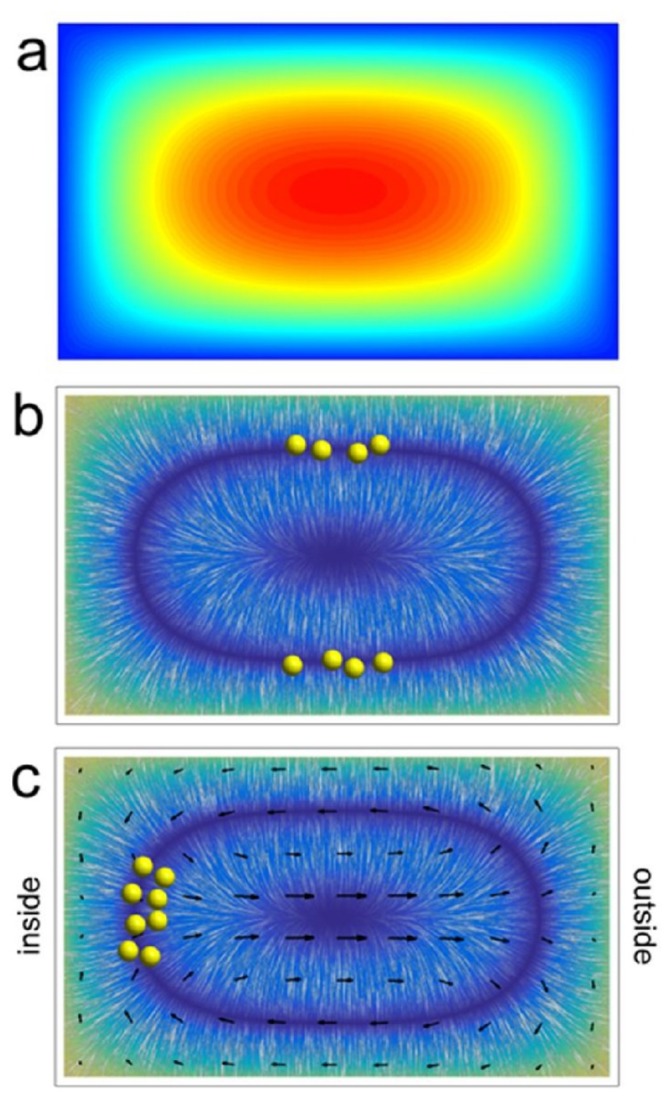
Inertial focussing of particles in microfluidic channels (qualitative). The coloured maps represent (a) the downstream fluid speed U and (b and c) the magnitude of the net lift force F_NL_; see Appendix A. The oriented texture superimposed on the latter is generated via line integral convolution [52], and is thus tangential to F_NL_. Particles (represented by yellow spheres) are driven away from the core of the flow toward regions of high stress, but are simultaneously kept away from the walls by asymmetric wake effects. In a straight rectangular channel (panels a and b), competition between these lift forces ultimately leads to particle aggregation along streamlines near the middle of the broad walls. In a curved channel (panel c), the induced secondary flow (indicated by arrows) contributes to the net lateral force and drives particles toward an apparent streamline near the midpoint of the inner wall of the turn. Here, and in all subsequent figures, the low end of the relevant scale (speed, force, etc) is mapped onto the colour blue.

Particles entrained in a steady laminar flow of fluid through a straight microfluidic channel experience inertial focusing effects. They are driven outward, away from the centreline of the channel, by gradients in the velocity U of the primary or downstream flow (Fig 1A) [44]. The magnitude of the lateral shear-induced lift force (F_SL_) to which they are subjected is of the form [45–49]

$${\text{F}}_{\text{SL}}={\text{f}}_{\text{SL }}\frac{\rho {\bar{\text{U}}}^{2}{\text{d}}^{4}}{{\text{D}}_{\text{h}}^{2}}+\text{O}\left({\text{d}}^{5}\right)$$ (1)

where ρ is the fluid density, $\bar{\text{U}}$ is its mean speed, d is the particle diameter, and D_h_ is the hydraulic diameter of the channel. The parameter f_SL_ is a dimensionless geometry-dependent field that encodes the net body force arising from viscous stresses on the particle and its associated flow field [45, 49]. This outwardly directed force is opposed by an inwardly directed “wall effect” lift force (F_WL_) caused by the asymmetric wake of the particle. The net lift force (F_NL_) varies in strength and direction over the channel cross section (Fig 1B), and is a function of particle size for given flow conditions [40, 47, 49, 50]. Within the core of the flow F_NL_ is dominated by F_SL_; near the walls it is dominated by F_WL_. Between these limits the net lift force acting on particles is much weaker than either F_SL_ or F_WL_ alone. The residual force in this intermediate region is largely tangential to the nearest wall, and is directed toward the channel midplane bisecting that wall [49, 51]. This feature is not shown in Fig 1B and 1C, as explained below. Overall, the net lift force causes particles to migrate across streamlines and collect along well-defined (size-dependent) lines of equilibrium on these midplanes where F_NL_ = 0. In a square channel particles of a given size are driven toward one of four lines of stable equilibrium parallel to the flow, each one near the midpoint of a wall. In a rectangular channel (lacking the fourfold symmetry of the square), the number of lines of stable equilibrium is reduced, and the tendency is for particles to collect near the midpoint of the broad walls where viscous shear is more intense (cf. Fig 1B).

Our characterization of the net lift force F_NL_ in Fig 1, and in subsequent figures, is approximate. It is based on an empirical mapping of the solution to the particle migration problem reported by Schonberg and Hinch [53], for Poisseuille flow between infinite parallel plates. The details of this mapping are described in Appendix A. This simple model captures key features of the inertial particle migration problem and provides a useful foundation for building qualitative understanding of the combined influence of hydrodynamic and magnetostatic forces on particle migration in curved channels. At the same time it should not be misconstrued as being a complete description of the problem. For example, our model clearly neglects the weak but important rotational interactions responsible for driving particle motion parallel to the walls in a straight channel [49, 51]. Thus F_NL_ = 0 in Fig 1b everywhere along a closed curve (the conspicuous dark blue oval) rather than at a finite number of discrete points along this curve, as occurs in practice and as implied by the clusters of particles that have been drawn. This omission would likely be significant if our goal was the study of inertial migration in straight channels. Fortunately these weak interactions tend to be dwarfed by the effects discussed next.

**Fig 2:**
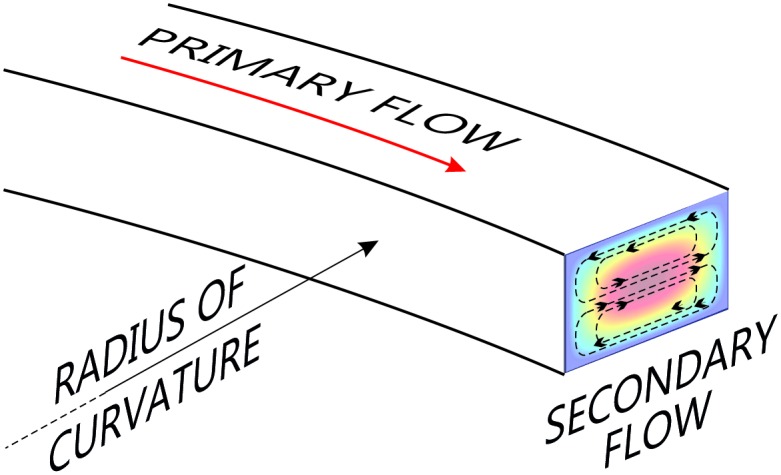
Geometry of a curved microfluidic channel, illustrating the relationship between the primary (downstream) and induced secondary flows.

The scenario outlined above is modified when the channel is curved (Figs 1C and 2). A recirculating secondary flow consisting of two counter-rotating vortices develops when fluid flows through a channel with a finite radius of curvature. The sense of this flow is such that it is directed outward (away from the centre of curvature) along the midline of the channel and inward near both of the walls parallel to the midline. This inertial effect is known as Dean flow [35, 36]. If particles are entrained in the primary flow, they are also influenced by the induced secondary flow. The drag force caused by the secondary flow can modify or disrupt the lines or surfaces of equilibrium where the net lateral force is zero, and along which inertial focussing of particles occurs.

The influence of channel curvature on flow is characterized by the dimensionless Dean number

$$\text{De}=\frac{\bar{\text{U}}{\text{D}}_{\text{h}}}{\nu }\sqrt{\frac{{\text{D}}_{\text{h}}}{2\text{ R}}}$$ (2)

where ν is the kinematic viscosity of the fluid and R is the radius of curvature. It depends on the primary downstream flow (through the Reynolds number $\text{Re}=\bar{\text{U}}{\text{D}}_{\text{h}}/\nu$) and the non-dimensional channel curvature δ = D_h_/(2R). To leading order in δ, and in the low Dean number limit, the amplitude U_D_ of the induced (transverse) rotational velocity field is expected to scale as

$${\text{U}}_{\text{D}}=\text{k De }\bar{\text{U}}\sqrt{\delta }=\text{k }\frac{\nu }{{\text{D}}_{\text{h}}}{\text{ De}}^{2}=\text{k }\frac{{\text{D}}_{\text{h}}^{2}}{2\nu \text{R}}{\bar{\text{U}}}^{2}.$$ (3)

Here the factor k relating the magnitude of the Dean flow velocity U_D_ to $\bar{\text{U}}$ is another dimensionless geometry-dependent field. Its maximum value lies in the range 10^−2^ to 10^−1^ for channel aspect ratios of order 1, and is equal to 3.4×10^−2^ for a square duct [54]. The magnitude of the ensuing lateral drag or “Dean force” exerted on particles that are stationary with respect to the channel cross section is then given by Stokes law:

$${\text{F}}_{\text{DL}}=3\;\pi \;\nu \;\rho \;\text{d}\;{\text{U}}_{\text{D}}.$$ (4)

This force should not be confused with the lateral drag experienced by particles that are entrained in the secondary flow, and thus have finite transverse velocities. The direction of the Dean force as defined above mirrors the rotational velocity field and its intensity scales as ${\bar{\text{U}}}^{2}$. Its tendency is to destabilize or eliminate lines of stable equilibrium for particle collection that are exhibited by flows in straight channels, except near the inner wall of the turn. Here a stable point is established where there is no net lateral force (the vector sum of F_NL_ and F_DL_). As $\bar{\text{U}}$ is increased, particles are observed to cluster near the midpoint of the inner wall and are transported along an apparent streamline of the downstream flow [38, 40, 43]. Importantly, the balance between the shear-induced lift and Dean forces (cf. Eqs 1 and 4) is strongly dependent upon particle diameter d, and so this clustering effect can and has been be employed in efficient continuous size-dependent fractionation of particle suspensions.

In detail, the problem of inertial focussing in curved channels is more complex and nuanced than the simple picture outlined above [55, 56]. This is particularly true as the Dean and Reynolds number of the flow are increased, or as the particle diameter increases to the point where finite size effects (such as perturbation of the underlying flow) can no longer be ignored. In such cases the apparent foci for aggregation of particles of a given size can transition from the inner wall of the turn to a point or points that are closer to the outer wall, accompanied by a shift away from the midline of the channel. And, at yet higher Dean numbers, more complex secondary flow patterns are known to develop. Even in the low Dean number limit, where experiments reported in this contribution are performed, a comprehensive predictive model for particle dynamics and inertial focussing phenomena in curved channels has yet to be developed.

### 1.2 Magnetic forces

The magnetic force acting on an MMS is proportional to its magnetic moment m, which in turn may be a function of applied magnetic field H. Following Ref. [5] we consider two limiting cases: that in which m = |m| increases in proportion to H = |H| and that in which |m| is independent of |H|. We refer to these idealizations as the unsaturated and saturated material responses, respectively, and note that our primary focus here is on the unsaturated case. That is, situations in which the magnetic response of the MMS is adequately described by a linear induced magnetic moment. With the further assumptions that the MMS is a sphere of diameter d and that the distribution of magnetic material within the particle is uniform, the magnitude of the force it experiences when exposed to a non-uniform field H is

$${\text{F}}_{\text{m},\text{u}}=\frac{\mu_{0}{\pi \chi \text{d}}^{3}}{4}\left|\nabla {\text{H}}^{2}\right|,$$ (5)

where μ_0_ is the permeability of free space. Here *χ*>>1 is the effective magnetic susceptibility of the particle. The equivalent expression for the limit in which the magnetic response of the particle is saturated is

$${\text{F}}_{\text{m},\text{s}}=\frac{\mu_{0}{\pi \text{M}}_{\text{s}}{\text{d}}^{3}}{6}\left|\nabla \text{H}\right|,$$ (6)

where M_s_ is the effective saturation magnetization of the particle. In both cases the factor of d^3^ comes from the proportionality of the force to m and hence to the volume of the particle. Appropriate modifications to these expressions are required if the magnetic and hydrodynamic diameters of the particles are different.

#### Interplay between magnetic and hydrodynamic forces

**Fig 3:**
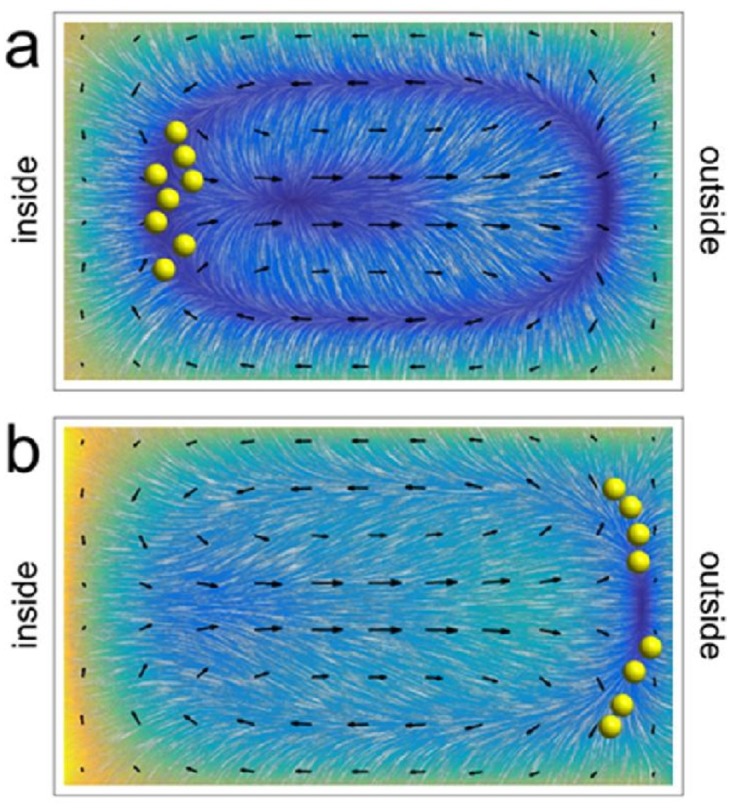
Interplay between magnetic and hydrodynamic forces acting on MMS in a curved channel (qualitative). The coloured maps and oriented texture are analogous to those shown in Fig 1B and 1C. They represent the vector sum of the magnetostatic, shear, and wall forces experienced by entrained particles. Secondary (Dean) flow is indicated by arrows. When the magnetic force is weak (panel a), particles aggregate near the inside wall (cf. Fig 1C). When it is strong (panel b), they are drawn toward the outside wall. The magnetic force is directed radially outward in both cases, and the downstream flow rate is the same as in Fig 1. The magnetic force applied in the example shown in panel (b) is five times as strong as that in panel (a).

The direction of the magnetic force exerted on a magnetic particle is governed by the direction of the applied magnetic field gradient, through either ∇H^2^ ≡ 2(H ·∇)H or ∇H (cf. Eqs 5 and 6). In our work with flow in curved microfluidic channels we have chosen to set the direction of this force so that it points radially outward from the centre of the spiral. It thus contributes to the net lateral force experienced by particles entrained in the downstream flow and can influence the inertial focussing to which they are subjected, as illustrated in Fig 3. Note that the magnitude of the magnetic force applied in our experiments is essentially uniform over the cross section of the channel.

Inertial focussing will occur near the inside wall of the turn, as usual, when the magnetic force is weak compared to other lateral forces acting on entrained particles (Fig 3A). In particular, this equilibrium is stabilized by secondary flow converging toward the midline of the channel. We expect this equilibrium to destabilize as the magnitude of the magnetic force is increased to the point where it becomes comparable to Dean drag forces. In its place we expect a new equilibrium will form near the outer wall of the turn (Fig 3B). The symmetry of the secondary flow pattern suggests that MMS may (or may not) cluster around two equivalent lines of equilibrium near the corners of the channel, depending on the relative strength of this flow. As a function of increasing flow rate (as opposed to increasing magnetic force), we expect a transition from a regime in which particles cluster near the outer wall of the turn to one in which they cluster near the inner wall of the turn. And, comparing Eqs 1, 4 and 5 (or Eq 6), we expect that the transition from one focussing regime to another will depend on particle diameter.

## 2. Experimental

### 2.1 Microfluidic spiral

**Fig 4:**
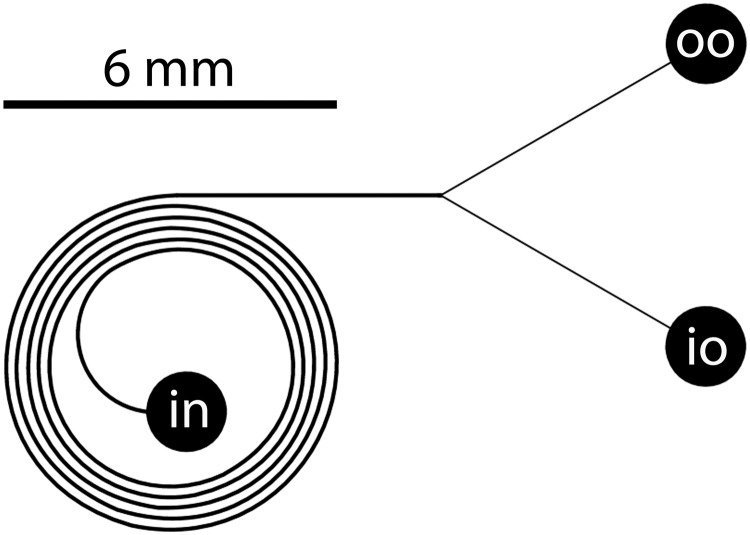
Geometry of the microfluidic spiral, as described in the text.

The microfluidic system employed in our experiments is shown schematically in Fig 4. The geometry is based on parameters evaluated during an earlier investigation [43] in which efficient fractionation of MMS with diameters spanning the range 2–15 μm was demonstrated. The channel consists of a 5-turn Archimedean spiral in a polydimethylsiloxane (PDMS) substrate. The innermost and outermost radii of this spiral are 2 and 3 mm, respectively. The channel is rectangular in cross section, with a width w = 100 μm in the plane of the spiral and a height h = 60 μm. These dimensions yield a hydraulic diameter D_h_ = 2wh/(w + h) of 75 μm and non-dimensional curvatures δ in the range 1 to 2%.

Fluid is injected via an input port (in) located near the centre of the spiral. It exits via a symmetric flow splitter and two outlet ports. The splitter is balanced 1:1 and separates the flow into two volumetrically equal streams. We refer to the exit ports as the “inner outlet” (io) and “outer outlet” (oo) based on their position relative to the spiral structure and their intended functions. That is, the role of the inner and outer outlets is to extract the inner and outer halves of the fluid stream exiting the spiral, respectively. Further details regarding the preparation and operation of these microfluidic chips can be found in [43].

### 2.2 Magnetic field gradient

The design of the magnetic field gradients used in our experiments is motivated by a multipole expansion of the field produced by sources exterior to the region of interest. Expressed in cylindrical coordinates, a two-dimensional multipolar magnetic field of order 2n satisfies

$$\text{H}={\text{H}}_{0}{\left(\frac{\text{r}}{\text{R}}\right)}^{\text{n}-1},$$ (7)

where H_0_ is the magnitude of the field at radius r = R. Consequently,

$$\nabla {\text{H}}^{2}=\left(2\text{n}-2\right){\text{H}}_{0}^{2}\frac{{\text{r}}^{2\text{n}-3}}{{\text{R}}^{2\text{n}-2}}\hat{\text{r}}$$ (8)

and

$$\nabla \text{H}=\left(\text{n}-1\right){\text{H}}_{0}\frac{{\text{r}}^{\text{n}-2}}{{\text{R}}^{\text{n}-1}}\hat{\text{r}}$$ (9)

are both directed radially outward from the axis of symmetry as indicated by the unit vector $\hat{\text{r}}$, and as required for the channel geometry discussed in the previous section.

**Fig 5:**
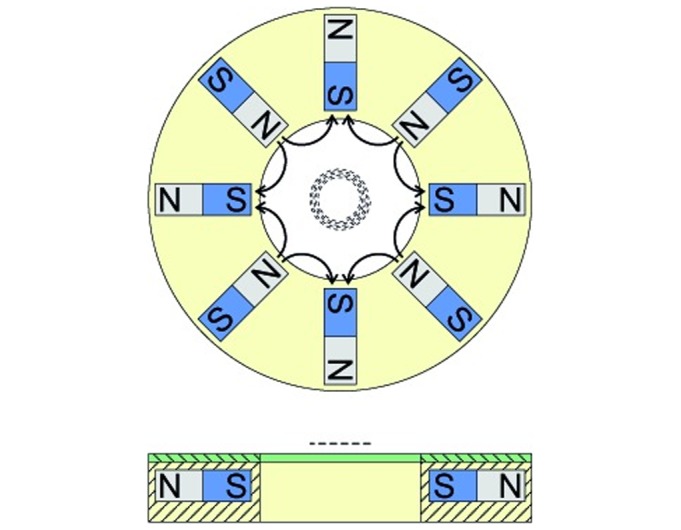
Schematic plan and (cross-sectional) side views of the array used to produce an octupolar magnetic field. Each 3.2 mm × 3.2 mm × 9.5 mm NdFeB magnet is magnetized parallel to its long axis. The distance between faces of opposing magnets is 17.7 mm and the diameter of the accessible bore is 16 mm. Curved arrows shown in the plan view indicate the qualitative sense of magnetic field lines. A thin annular ferromagnetic shield (green; side view only) between the array and the plane of the microfluidic spiral screens the magnetic field in the vicinity of the outlet ports and provides a field-free region for particle extraction. The distance of closest approach between the microfluidic spiral (represented by the dashed curve/line) and the plane of the shield is set by a microscope slide (not shown). See also Fig 6.

**Fig 6:**
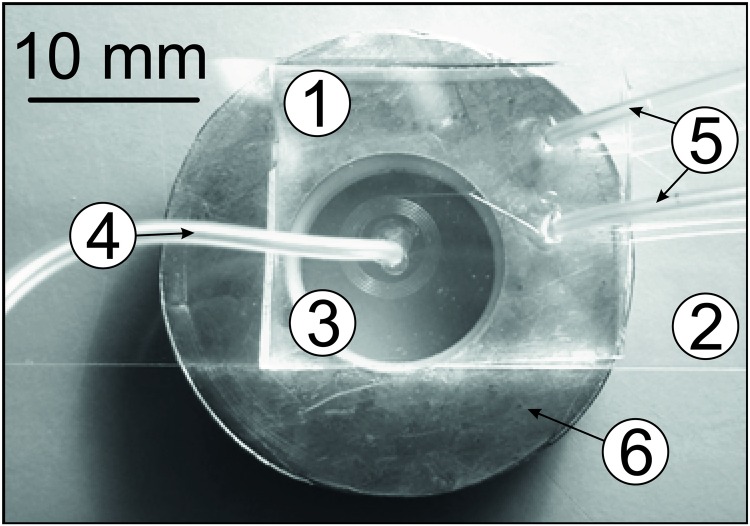
Photograph of the microfluidic chip (1) on a microscope slide (2) showing the spiral structure (3), inlet (4), and two outlets (5). The array of permanent magnets is not visible; it is located beneath the soft ferromagnetic shield (6). Note that the spiral is accurately centred with respect to the magnetic axis during operation; the apparent shift in location is an artefact of the angle from which the photograph was taken.

In practice, we employed a planar array of NdFeB permanent bar magnets arranged as shown in Fig 5. The magnetic field near the centre of this array has excellent octupolar (2n = 8) symmetry, and thus |∇H^2^| and |∇H| scale as r^5^ and r^2^ (cf. Eqs 8 and 9), respectively. This array was positioned adjacent to (and coaxial with) the microfluidic spiral (cf. Figs 5 and 6). In this way the intensity of the radial component of the magnetic field gradient can be adjusted by varying the distance between the plane of the array and the plane of the spiral.

A feature of this design is the fact that the magnetic field near the centre of the array (and hence the centre of the microfluidic spiral) is uniform. This eliminates magnetic forces and facilitates MMS injection through a 0.5 mm diameter tube into the spiral (Fig 6). In particular, it precludes the agglomeration of particles and subsequent occlusion of the centrally-located injection port, as was observed previously when inhomogeneous magnetic fields were applied [43]. Efficient extraction of MMS from the microfluidic channel is then accomplished by placing a 0.8 mm thick galvanized sheet steel disc between the magnet array and the plane of the spiral, as shown in Figs 5 and 6. This soft ferromagnetic shield provides a low reluctance path between the two ends of each magnet, and provides a well-screened region of space nearby in which magnetic field gradients are supressed, and in which the outlet ports can be safely located. Importantly, the shield retains the symmetry of the field to which MMS in the spiral are exposed.

**Fig 7:**
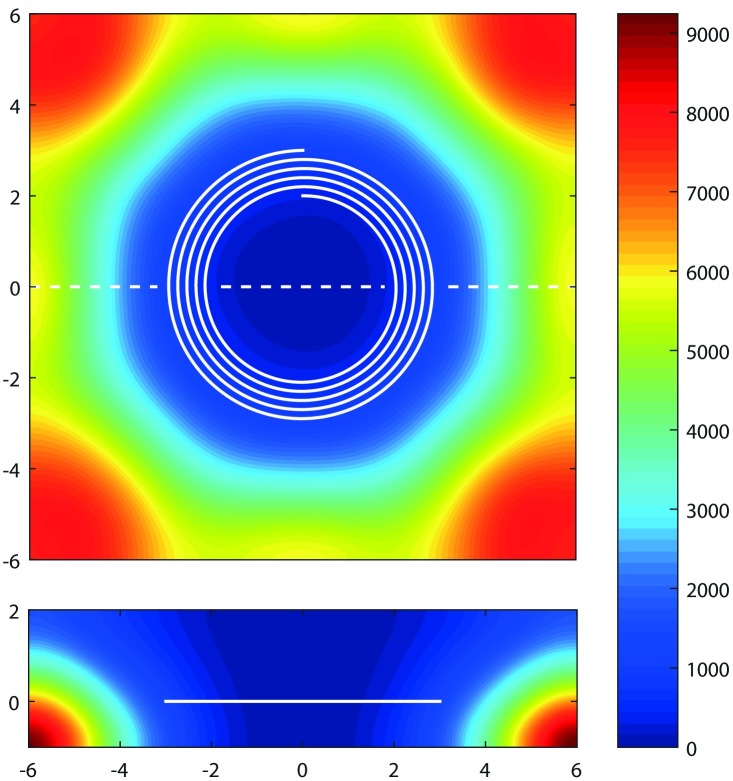
Calculated map of the magnetic field strength H, in units of A/m. The microfluidic channel is shown as a white spiral (upper panel; plan view) and a white line (lower panel; side view). Spatial coordinates are specified relative to its centre, in units of mm. The dashed line indicates the intersection of the two orthogonal views.

Fig 7 shows a map of H in the vicinity of the microfluidic spiral, as calculated using an experimentally validated numerical model. The maximum field to which isolated MMS are exposed along their trajectory through the spiral is of order 10^3^ A/m, which is well below core saturation conditions. Eq 5 thus gives the appropriate expression for the magnetic force to which isolated particles are subjected, in terms of |∇H^2^|. Empirically we find that the field in the vicinity of the spiral is well characterized by |∇H^2^| = (1.2 × 10^22^ A^2^/m^8^)r^5^. This gradient is generally radial in direction as desired, but does have vertical and azimuthal components as can be inferred from the figure. This gradient increases by a factor of about four when the ferromagnetic shield is removed.

### 2.3 Magnetic microspheres, suspensions, and flow conditions

A variety of MMS were employed in inertial focussing experiments. In some cases, MMS with diameters of 2, 6, or 12 μm were used. These particles comprise a styrene-maleic acid copolymer matrix encapsulating 50% by mass magnetite cores (MMS density = 1.85 g/cm^3^; Micromer^®^-M; Micromod Partikeltechnologie, Rostock, Germany). The mean low-field susceptibility, coercive field, and saturation magnetization of the particle cores are χ = 92(14), H_c_ = 3.17(1) × 10^3^H_c_ A/m, and M_s_ = 1.7(1) × 10^5^ A/m, respectively, as measured using a vibrating sample magnetometer (Micromag 3900, Princeton Measurement Corporation, Princeton, NJ, USA). In other cases, specially prepared MMS with a broad distribution of sizes (spanning the range 0.3 μm to 12 μm, with a volume averaged mean of 3.5 μm) were used. The latter comprise a silica matrix with silanol groups on the surface encapsulating 50% by mass maghemite cores (MMS density = 2.25 g/cm^3^; SiMAG-Silanol, Chemicell, Berlin, Germany).

Aqueous suspensions with MMS concentrations of 0.5% by mass were injected from a disposable syringe into the microfluidic channel at flow rates ranging from 5 to 80 μL/min controlled by a BS-8000 syringe pump (Braintree Scientific, Braintree, MA, U.S.A.). The average downstream flow speed, Reynolds number, and maximum Dean number corresponding to the maximum flow rate employed were therefore U = 0.22 m/s, Re = 17, and De = 1.6. Suspensions extracted from the inner- and outer-outlets were collected and characterized. Photographs of MMS distributions were acquired using a camera (Infinity C1-3, Mazurek Optical Services, Southam, UK) that viewed the spiral through a microscope (AE31, Motic, Hong Kong, China).

The behaviours of MMS suspensions with the following compositions were investigated:

1. MMS with a uniform diameter of 6 μm; The number of MMS extracted from both outlets was determined using a haemocytometer (Hausser Scientific, Horsham, PA, USA), and was studied as a function of flow rate.
2. A bimodal 1:1 mixture of MMS with uniform diameters of 2 μm and 12 μm; The relative number of MMS in samples extracted from both outlets was determined using static light scattering (Mastersizer 2000, Malvern Instruments, Malvern, UK), and was studied as a function of flow rate.
3. MMS with a disperse distribution of diameters, and a mean diameter of 3.5 μm; Mean particle diameters and distributions in samples extracted from both outlets were characterised by static light scattering, and were studied as a function of flow rate.

In all three cases the applied magnetic field gradient in the vicinity of the spiral was held constant as the flow rate was changed.

Checks were made to ensure that volume weighted particle size distributions inferred from light scattering experiments were not influenced by size-dependent scattering effects. This involved working with bimodal mixtures of uniformly sized MMS and constructing appropriate sums and ratios of peak areas obtained from samples extracted from the inlet and the two outlets. Comparisons of inferred particle ratios were also checked against known particle concentrations in the stock solutions.

## 3. Results and Discussion

### 3.1 Uniform MMS

**Fig 8:**
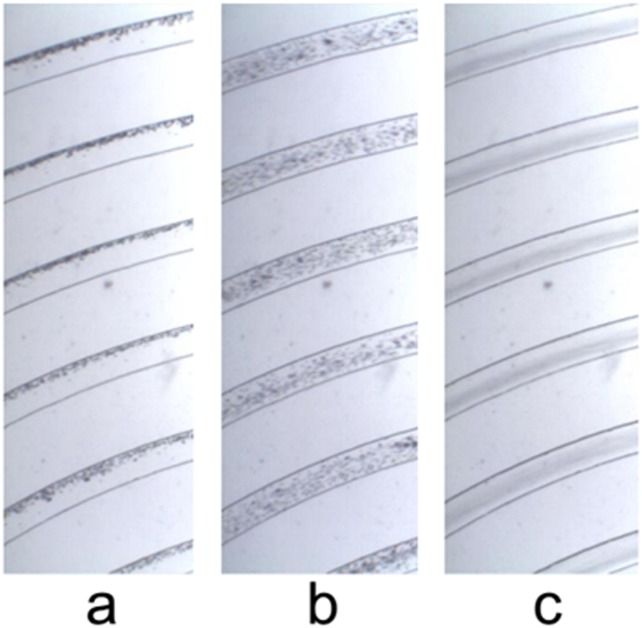
Photographs of MMS distributions in the microfluidic spiral at (a) low (5 μL/min), (b) medium (30 μL/min), and (c) high (60 μL/min) flow rates. The view is from above the spiral, looking down toward the magnet array. The full vertical extent of the channel lies within the optical depth of field.

**Fig 9:**
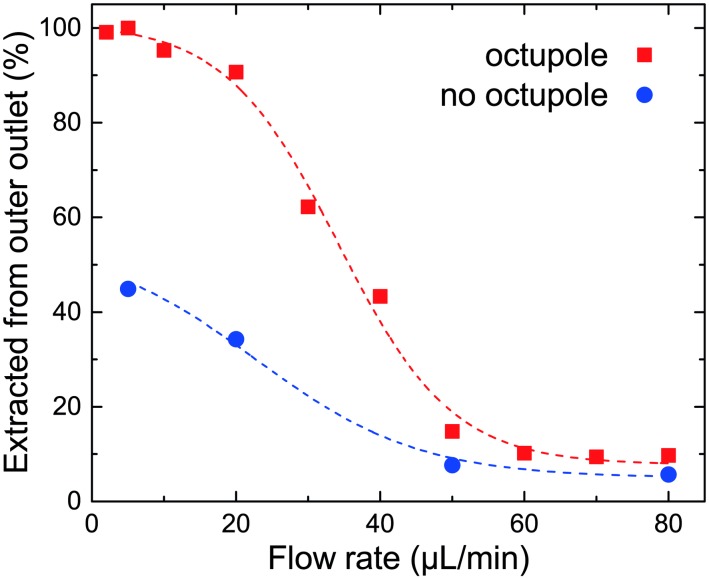
Fraction of 6 μm diameter MMS extracted from the outer outlet, as collected at different flow rates. The remaining MMS are extracted from the inner outlet. Similar data acquired without the octupole [43] are shown for reference. The curves are only intended as guides for the eye.

At low flow rates (5 μL/min) we observe that 6 μm diameter MMS aggregate along an apparent streamline near the outer wall of the spiral. At intermediate flow rates (30 μL/min) the same MMS are not focussed; they are homogeneously distributed across the width of the channel. At high flow rates (60 L/min) they are once again focused; but this time they aggregate within the inner-half of the spiral. This behaviour is evident both in photographs of MMS flowing through the spiral (Fig 8) and in measurements of the relative number of particles extracted from the two outlet ports (Fig 9). Note that while all of the MMS exit through the outer outlet at low flow rates, only 90% of them exit through the inner outlet at the highest flow rate investigated.

This behaviour is consistent with the model presented in section 1. We infer that Dean drag forces play little or no role in establishing the low flow rate equilibrium for MMS near the outer wall of the spiral; instead this lateral equilibrium is determined by competition between magnetostatic, lift, and wall forces. As the downstream flow rate increases, so does the secondary flow rate. Eventually drag forces associated with the inwardly directed component of the secondary flow overcomes magnetostatic forces and MMS are pushed toward the inner half of the channel. Here a new equilibrium is established. Between these limits, competition between hydrodynamic and magnetostatic forces leads to a situation where efficient lateral focusing does not occur on the length scale of the spiral.

**Fig 10:**
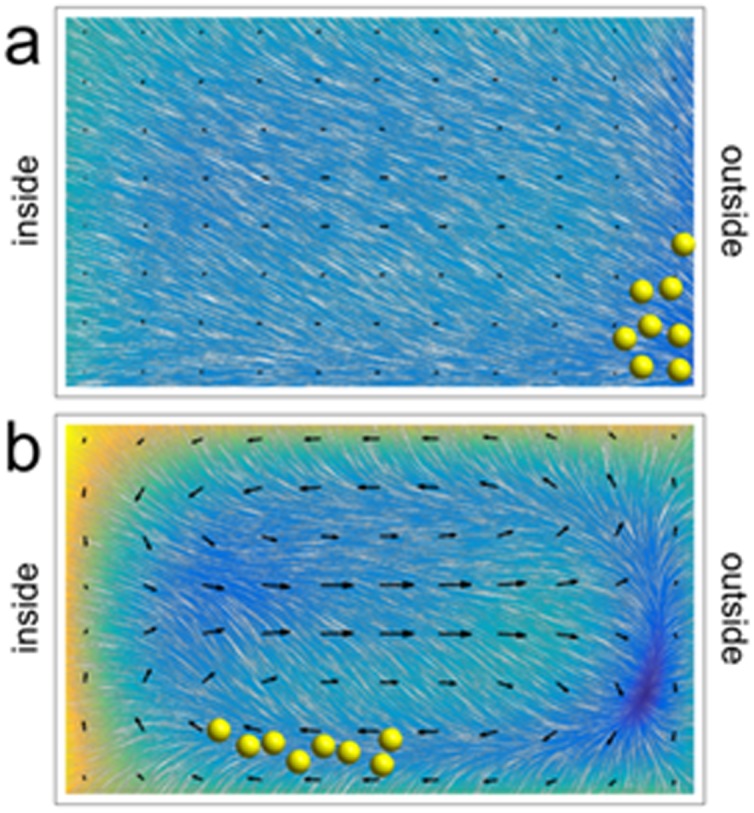
Qualitative interpretation of MMS focussing data at low (panel a; cf. Fig 8A) and high (panel b; cf. Fig 8B) flow rates. The coloured maps are analogous to those shown in Fig 3. That is they represent the magnitude of the net lateral force acting on particles excluding the drag associated with the induced secondary flow. The latter is represented by arrows. The outwardly-directed magnetic field gradient is canted downwards by 25 degrees relative to the horizontal to reflect experimental conditions.

The data from these experiments are interpreted qualitatively in Fig 10. This figure shows scenarios similar to those presented in Fig 3, except the downstream flow rate is changed instead of the magnetic field gradient. Additionally, the outwardly-directed magnetic field gradient is canted downward by 25 degrees relative to the horizontal to better reflect experimental conditions. Recognizing that the downward component of the magnetostatic force is supplemented by the gravitational force, it is likely that particles preferentially collect near the bottom outside corner of the channel at low flow rates (cf. Figs 8a and 10a). And, it is likely that the focussed state identified in Fig 8C corresponds to MMS localization along the inner part of the bottom wall of the channel (cf. Fig 10B) rather than near the midpoint of the inner wall (cf. Fig 3B). The latter inference is consistent with the observation that some MMS exit through the outer outlet even at the highest flow rates studied (cf. Fig 9). It also hints at the possibility that gravitational forces may have contributed to the focussing of at least some MMS near the inner half of the lower wall during experiments performed previously without magnetic field gradients [43].

A unique feature of these experiments is that they suggest a method for quantitative in-situ measurement of Dean drag forces. As an illustration of how this information can be extracted from the data, consider the following arguments based on simple estimates. First, the manufacturer’s specified density for the MMS used in these experiments relative to that of stoichiometric magnetite imply an equivalent core diameter of 3.4 μm. When combined with the measured core susceptibly and applied field gradient ∇H^2^, we infer from Eq 5 that an isolated MMS experiences an average outwardly-directed magnetostatic force F_m,u_ of 6 pN as it travels through the spiral. An equilibrium, such as those suggested in Figs 3B and 10A (or apparent in Fig 8A), will persist until the inwardly-directed component of the drag force associated with the local secondary flow exceeds F_m,u_. For a 6 pN force acting on a 6 μm diameter particle in water, this occurs when the magnitude of the inwardly-directed component of the local Dean velocity exceeds U_D_ = 1 × 10^−4^ m/s (cf. Eq 4). Next, consider the data shown in Fig 9 which indicates that particles are equally-likely to be extracted from either of the two outlets at a flow rate of about 35 μL/min, where the average downstream flow speed $\bar{\text{U}}\;=\;0.1$ m/s. If we interpret this condition as a signature of the point at which the magnetostatic force is counterbalanced by Dean drag (and hence the local value of F_DL_ = 6 pN), the implied (experimentally determined) relationship between the local value of U_D_ and $\bar{\text{U}}$ (cf. Eq 3) is ${\text{U}}_{\text{D}}=0.009\;{\text{D}}_{\text{h}}^{2}\bar{\text{U}}2/\left(2\nu \text{R}\right)$. This result is reasonable and consistent with expectations. For context, it amounts to about one-quarter of the maximum theoretical secondary flow speed induced in a square channel [54]; or, about one-half of the secondary flow speed near the walls of that same square channel. At the same time, one should recognize this result for what it is: an estimate that relies on several approximations. One could certainly imagine pursuing refinements to this experiment in which systematic variation of the applied magnetostatic force is combined with computational modelling of fluid and particle dynamics to extract Dean drag forces and flow speeds over a wide range of conditions.

### 3.2 Bimodal mixture of uniformly sized MMS

**Fig 11:**
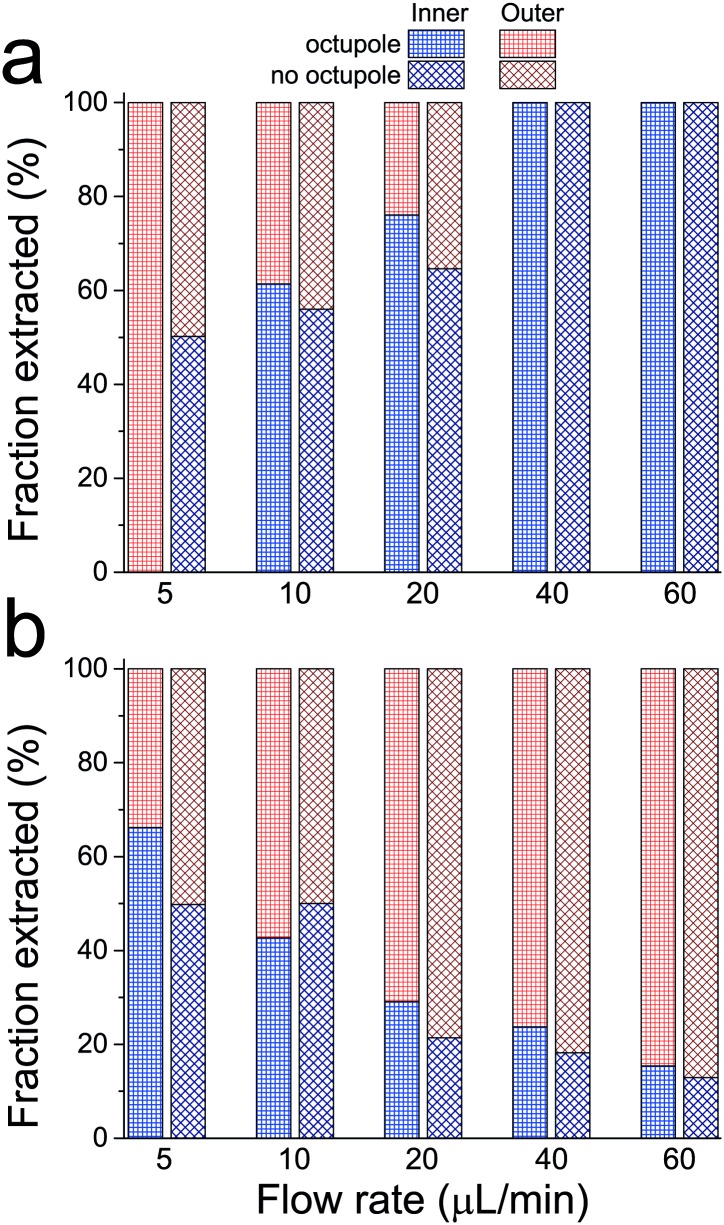
Fraction of (a) large (12 μm diameter) and (b) small (2 μm diameter) MMS extracted from the two outlets during experiments in which a 1:1 mixture of the two components was injected into the spiral. Data acquired without the octupole [43] are shown for reference.

At low flow rates (5 μL/min) we observe that all of the 12 μm diameter MMS and approximately one-third of the 2 μm diameter MMS are drawn toward the outer wall of the spiral, and are extracted from the outer outlet as indicated in Fig 11. The remaining two-thirds of the 2 μm diameter MMS are extracted from the inner outlet. As was the case for the 6 μm diameter MMS studied above, progressively more 12 μm diameter MMS are extracted from the inner outlet as the flow rate is increased. By the point at which the flow rate has reached 40 μL/min, all of the 12 μm diameter MMS exit through the inner outlet. The behaviour of the 2 μm diameter MMS is opposite that of the larger particles; fewer and fewer are extracted from the inner outlet as the flow rate is increased.

These data exhibit features that depart from the simple model of inertial focussing considered to this point. The larger particles can be efficiently drawn toward the outer half of the fluid stream by magnetostatic forces at low flow rates, or to the inner half of the fluid stream by hydrodynamic forces at high flow rates. However, the transition from one regime to the other occurs at a much lower flow rate than it does for the 6 μm diameter particles considered previously. In fact, it is only at the lowest flow rate shown in Fig 11 that a dramatic effect associated with the applied magnetostatic force is even evident. One would naively expect the magnetostatic equilibrium for 12 μm diameter particles exiting via the outer outlet to be more stable against perturbation by Dean flow than for 6 μm diameter particles, rather than the opposite. The explanation for this unanticipated trend may be related to the horizontal component of F_NL_ caused by fluid shear acting in concert with the secondary flow. It might also be linked to finite particle size effects which can influence inertial focussing [46, 50, 56, 57]; in this context, note that 12 μm is equivalent to 20% of the channel height in our experiments. In either case, computational modelling of fluid and particle dynamics would likely be required to gain further insight.

As for the 2 μm diameter MMS, the fact that they cluster near the outer wall of the turn (rather than the inner wall) at high flow rates is also not explained by the simple picture of inertial focussing presented in section 1. Nevertheless, it is evident that the gross inertial focussing behaviour of these particles is dominated by hydrodynamic effects, as can be seen by comparing data acquired with and without the octupole array. This is to be expected, and is consistent with our simple model. For small geometrically similar MMS the relative importance of magnetostatic and Dean drag forces scales as particle diameter squared (cf. Eqs 4 and 5). Consequently, the role of magnetostatic forces in the focussing of 2 μm diameter particles ought to be an order of magnitude less important than it is for 6 μm diameter particles. Indeed, the only clear signature of a magnetostatic effect on the focussing of 2 μm diameter particles is the somewhat elevated fraction extracted from the outer outlet at the lowest flow rate, relative to the 50% that are expected (and observed) without the octupole array.

### 3.3 MMS with a broad size distribution

**Fig 12:**
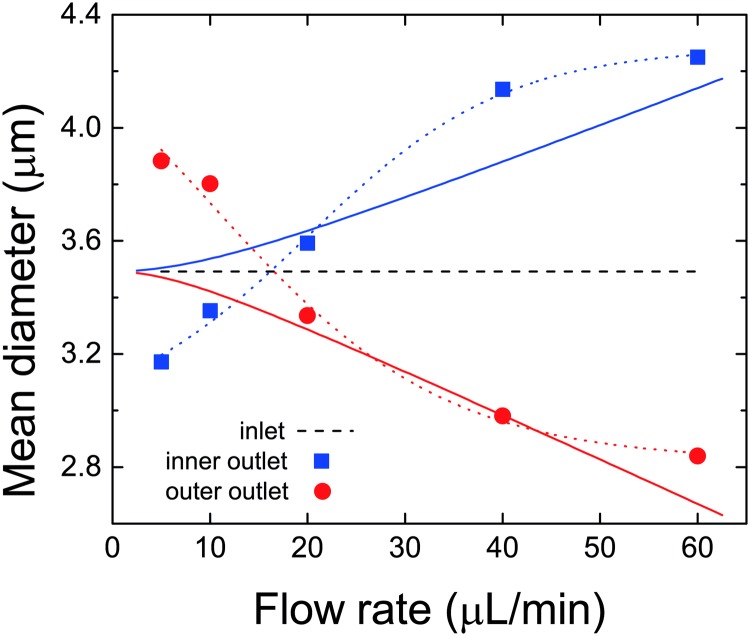
Volume-averaged diameters of disperse MMS extracted from the two outlet ports of the spiral (symbols). Also shown for comparison are the mean diameter of MMS injected into the spiral (dashed line), and the nearly-linear trends in the deviation of particle diameter from the mean that are observed when the applied field gradient is eliminated. The latter are plotted as solid lines (red: outer outlet; blue: inner outlet) and replicate the deviation of particle diameters from the mean given in Fig 9 of [43] to better than 0.02 μm. The dotted lines are only intended as guides for the eye.

Fig 12 summarizes our experimental observations regarding the influence of an applied magnetic field gradient on the mean diameter of particles that are extracted from the two outlets of the microfluidic spiral when the stock suspension that is injected comprises MMS with a disperse distribution of diameters. Analysis of this suspension yields a mean volume-averaged diameter of 3.5 μm. At low flow rates we observe that the mean diameter of particles extracted from the outer (inner) half of the fluid stream is increased (decreased) relative to the mean diameter of the injected suspension. At high flow rates we observe the opposite; the mean diameter of particles extracted from the outer (inner) half of the fluid stream is decreased (increased) relative to the mean diameter of the injected suspension.

This behaviour reflects the general trends reported above for experiments with uniformly sized MMS. For example, at low flow rates we expect particles with larger diameters of order 6 μm and 12 μm to be preferentially extracted from the outer outlet, while particles with smaller diameters of order 2 μm are preferentially extracted from the inner outlet. And at high flow rates we expect the opposite to occur. In both cases these trends will cause the mean diameter of particles extracted at the two outlets to shift in the same sense as is observed.

Further insight into the roles played by magnetostatic and hydrodynamic forces in determining the outcomes of the complex interactions taking place during these experiments can be obtained by comparing the data to the two solid curves shown in Fig 12. These curves represent the nearly linear trends in particle diameter that are observed during analogous experiments performed on similar suspensions, but with no applied field gradients [43]. The most apparent difference between the data from the two experiments is the non-linear response that is observed when magnetostatic forces are introduced. Significant deviations between the two are evident, as expected, at low flow rates where the strength of the secondary flow field is weak. However, there is also a significant increase in the mean diameter of particles extracted from the inner outlet at flow rates of order 40 μL/min.

## 4. Conclusion

We have investigated consequences of applying an outwardly directed magnetic force to a suspension of magnetic microspheres circulating in a spiral microfluidic channel. That is, a force that opposes inertial focusing of particles near the inner wall of the turn. At low flow rates we find that a new regime of stable particle focussing is established near the outer wall of the turn. At high flow rates this new equilibrium is destroyed by secondary (Dean) flow and particles once again cluster near the inner wall of the turn, or within the inner half of the channel. The transition from one regime to another depends on particle size; in our experiments they were observed at Dean numbers De < 1.

General features of this new focussing regime and transitions to conventional particle suspension behaviour in curved channels are explained by a rudimentary model incorporating magnetic and hydrodynamics forces. At the same time, there are aspects of the behaviour we observe and report that are not encapsulated in this model, and which may be related to hydrodynamic interactions that have been ignored.

One of the most significant findings we report is a method for quantitative in-situ determination of Dean drag forces and velocities. In its simplest incarnation, and as demonstrated in our report, it involves establishing a balance between opposing magnetostatic and Dean drag forces and then examining particle responses as one or the other is changed. We anticipate that future systematic studies performed using this measurement technique combined with computational modelling of fluid and particle dynamics could provide a rich source of quantitative information about particle focusing in microfluidic channels.

More generally, our investigation informs understanding of the complex interactions to which particles are subjected in curved microchannels, and provides a foundation upon which new devices and/or processes might be developed. In particular, it suggests particle processing methods in which magnetic and hydrodynamic interactions in curved channels are independently tuned or controlled to achieve a desired outcome with higher fidelity or greater specificity. An example of a highly desirable outcome would be a scheme for efficient and continuous sorting, extraction or enrichment of particles with characteristics that are well-suited for therapeutic or diagnostic applications of MMS ([1, 58]).

In this context, note that while our experiments were performed using an external magnet array, this is not a general design requirement. One could certainly employ technologies ranging from onboard or integrated permanent magnet and electromagnet arrays to magnetic flux concentrators to realize similar or enhanced performance. Additionally, while the magnitude of the magnetic forces applied in our experiments varied as a function of radius, this is also not a general design requirement. One could equally well imagine designing an apparatus in which the magnitude of the magnetostatic force is independent of radius. (For that matter, there is no general requirement for the flow to be confined to a spiral. In this limit there are important connections between our experiments and conventional continuous flow magnetic separation or particle manipulation schemes; see Ref. [59] for examples. And, even though our experiments were performed in the limit where the MMS cores were unsaturated, this is again not a general requirement. Appropriate magnetic fields could be applied to the microfluidic channel to reach (or even modulate) desired operating conditions.

Finally, it is worth emphasizing that our apparatus was not designed to enhance MMS fractionation efficiency, but rather to study the interplay between magnetic and hydrodynamic forces; hence the choice of an outwardly directed magnetic force. If instead the goal is to enhance focussing efficiency, one would likely want to arrange the magnetic force so that it is directed radially inward and complements (rather than opposes) the hydrodynamic effect.

## Appendix

Figs 1, 3 and 10 show maps depicting lateral forces that contribute to establishing equilibrium distributions of particles entrained in steady laminar flows through microfluidic channels. These maps are qualitative. They are intended to provide insight into some of the experimental observations we report, and to motivate future investigations. They are not intended as a basis from which inferences about subtle details of particle migration behaviour can or should be drawn.

This appendix summarizes our treatment of the net lift force **F**_NL_, which acts perpendicular to the downstream flow and which contributes to the net lateral force experienced by particles. Various analyses of this problem have been reported for flow between or adjacent to infinite parallel planes (*e*.*g*., [45, 48, 53, 60]) and in square channels [49]. In principle we could have adapted the recent computations of **F**_NL_ performed by Hood *et al* for a square channel to the appropriate geometry and flow conditions. Instead, we approximate the net lift experienced by particles in our experiments via an empirical mapping of the results obtained by Schonberg and Hinch [53] for laminar flow between infinite parallel planes. Specifically, we map the one-dimensional coordinate s that characterizes the normalized position of a particle between infinite parallel plates onto contours of a two-dimensional (complex) normalized coordinate z = x + iy such that

$$\text{z}=\text{F}\left(\frac{2\text{D}}{{\text{D}}^{2}+1},\text{m}\right)/\text{K}\left(\text{m}\right)\;,$$ (A1)

where F and K are the incomplete and complete elliptic integrals of the first kind [61], respectively. Here

$$\text{D}={\left(1-2\text{s}\right)}^{\alpha }\frac{\left[\text{cos}\theta +\text{i sin}\theta \right]}{\sqrt{{\text{cos}}^{2}\theta +\frac{{\text{sin}}^{2}\theta }{{\left(1-\eta \right)}^{2}}}}$$ (A2)

for 0 ≤ *s* ≤ 1/2 and the parameter m satisfies IM[F(m^−1/2^,m)]/Re[F(m^−1/2^,m)] = h/w where h and w are the height and width of the channel, respectively. When s = 0 (corresponding to one of the two walls in the original one-dimensional problem) this mapping generates the periphery of the rectangle −1 < x <1; −h/w < y < h/w as θ is varied between–π and π; see for example [62]. As *s* approaches 1/2 (corresponding to the midpoint of the flow in the original problem) the mapping generates a vanishingly small elliptical contour of ellipticity *η*, at the midpoint of the rectangle. Between these limits the infinite strip −∞ < q = tanh^−1^(θ/π) < ∞; 0 < *s* < 1/2 in the complex plane w = q + is is mapped onto the interior of the rectangle. This mapping is parameterized by the exponent α and the ellipticity *η* appearing in Eq A2.

Empirical values for α and *η* are determined using Eqs A1 and A2 to map the normalized parabolic speed profile u = 1 − (1 − 2*s*)^2^ of the original problem onto the rectangle and then minimizing the rms deviation with respect to the normalized downstream flow speed for an incompressible Newtonian fluid in a straight channel

$$\text{U}\left(\text{x},\text{y}\right)={\left[\sum_{\text{n}=1}^{\infty }\frac{{\left(-1\right)}^{\text{n}}}{{\left(2\text{n}+1\right)}^{3}}\left(1-\frac{1}{\text{cosh}\left[\left(2\text{n}+1\right)\pi \text{w}/\left(2\text{h}\right)\right]}\right)\right]}^{-1}\cdot \left[\sum_{\text{n}=1}^{\infty }\frac{{\left(-1\right)}^{\text{n}}}{{\left(2\text{n}+1\right)}^{3}}\left(1-\frac{\text{cosh}\left[\left(2\text{n}+1\right)\pi \text{x}/\text{h}\right]}{\text{cosh}\left[\left(2\text{n}+1\right)\pi \text{w}/\left(2\text{h}\right)\right]}\right)\text{cos}\left[\frac{\left(2\text{n}+1\right)\pi \text{y}}{\text{h}}\right]\right]$$ (A3)

(cf. Fig 1A). No attempt is made to account for the influence of channel curvature on U as our focus here is on the small Dean number limit. Note also that the mapping outlined above is not expected to be exact; it merely yields an approximation to U. For a channel with aspect ratio h/w = 0.6 the rms deviation of the normalized downstream flow speed is reduced to 2% relative to Eq A3 for *α* = 0.51 and η = 0.52. Similar levels of agreement are obtained for channel aspect ratios over the range 0.5 < h/w < 1, yielding values of *α* and η as listed in “S1 Appendix”.

Once *α* and η are established, the net lift inferred from the solution presented by Schonberg and Hinch is mapped onto the appropriate rectangular channel, cf. Fig 1B. Again we emphasize that this mapping is empirical, and that it necessarily ignores contributions to the lift force that arise from coupling between particle dynamics and the finite transverse extent of the channel. Despite these shortcomings, it yields what is expected to be a reasonable depiction of F_NL_. For example, if the solution for **F**_NL_ presented by Hood *et al* for small particles in a square channel [49] is projected onto the map of **F**_NL_ generated as described above, and that projection is then used as a basis for comparison, the radial locations at which F_NL_ = 0 are reproduced by our mapping at the level of a few percent relative to channel dimensions. In fact, the general spatial distribution of F_NL_ given by the two maps is qualitatively very similar over the full central two-thirds of the flow (encompassing the regions where F_NL_ = 0). The only significant deviations between the two occur near the corners of the channel, where our mapping tends to overestimate F_NL_.

## Supporting Information

S1 Appendix Empirically-determined parameters *α* and η for channels of aspect ratio h/w. (DOCX)

S1 Data Dataset of performed measurements. (DOCX)
