# Supplementary material for: Fractionation of Magnetic Microspheres in a Microfluidic Spiral: Interplay between Magnetic and Hydrodynamic Forces
Source: PLoS One. 2017 Jan 20;12(1):e0169919. doi: 10.1371/journal.pone.0169919 (PMC5249185; doi:10.1371/journal.pone.0169919)
Supplement: S1 Appendix — (DOCX) [file pone.0169919.s001.docx]

SUPPORTING INFORMATION 1 - APPENDIX

**Fractionation of Magnetic Microspheres in a Microfluidic Spiral: Interplay between Magnetic and Hydrodynamic Forces**

S. Dutz ^1,2^*, M.E. Hayden ^3^, and U.O. Häfeli ^1^

^1^ Faculty of Pharmaceutical Sciences, University of British Columbia, Vancouver, Canada

^2^ Institute of Biomedical Engineering and Informatics (BMTI), Technische Universität Ilmenau, Ilmenau, Germany

^3^ Department of Physics, Simon Fraser University, Burnaby, Canada

| h/w | $\alpha$ | $\eta$ |
| --- | --- | --- |
| 0.5 | 0.35 | 0.66 |
| 0.6 | 0.51 | 0.52 |
| 0.8 | 0.79 | 0.24 |
| 1.0 | 0.98 | 0 |

Table S1: Empirically-determined parameters $\alpha$ and $\eta$ (cf. Eq. A2) for channels of aspect ratio $h/w$.
