# Supplementary material for: Fractionation of Magnetic Microspheres in a Microfluidic Spiral: Interplay between Magnetic and Hydrodynamic Forces
Source: PLoS One. 2017 Jan 20;12(1):e0169919. doi: 10.1371/journal.pone.0169919 (PMC5249185; doi:10.1371/journal.pone.0169919)
Supplement: S1 Data — (DOCX) [file pone.0169919.s002.docx]

SUPPORTING INFORMATION 2 - DATA

**Fractionation of Magnetic Microspheres in a Microfluidic Spiral: Interplay between Magnetic and Hydrodynamic Forces**

S. Dutz ^1,2^*, M.E. Hayden ^3^, and U.O. Häfeli ^1^

^1^ Faculty of Pharmaceutical Sciences, University of British Columbia, Vancouver, Canada

^2^ Institute of Biomedical Engineering and Informatics (BMTI), Technische Universität Ilmenau, Ilmenau, Germany

^3^ Department of Physics, Simon Fraser University, Burnaby, Canada

Table S2-1: Fraction of 6 μm diameter MMS extracted from the outer and the inner outlet, as collected at different flow rates.

Table S2-2: Fraction of 6 μm diameter MMS extracted from the outer outlet, as collected at different flow rates. The remaining MMS are extracted from the inner outlet. Similar data acquired without the octupole from previous study are shown for reference.

Table S2-3: Fraction of large (12 μm diameter) and small (2 μm diameter) MMS extracted from the two outlets during experiments in which a 1:1 mixture of the two components was injected into the spiral.

Table S2-4: Volume-averaged diameters of disperse MMS extracted from the two outlet ports of the spiral as collected at different flow rates
